# Parents’ Satisfaction with Juvenile Idiopathic Arthritis Care: Findings from a Cohort of Italian Children Using the JAMAR Questionnaire

**DOI:** 10.3390/medicina61061115

**Published:** 2025-06-19

**Authors:** Federica Romano, Precia Mombasi, Franco Garofalo, Nima Namarvari, Francesco Franco, Patrizia Defabianis, Giovanni Nicolao Berta

**Affiliations:** 1 Department of Surgical Sciences, C.I.R. Dental School, University of Turin, Via Nizza 230, 10126 Turin, Italy; federica.romano@unito.it (F.R.); mompasi20@gmail.com (P.M.); patrizia.defabianis@unito.it (P.D.); 2 Paediatric Rheumatology Unit, Paediatric Department, Rivoli Hospital, Via Rivalta 29, 10098 Rivoli, Italy; franco.garofalo37@gmail.com; 3 Department of Clinical and Biological Sciences, Section of Translational Pharmacology, University of Turin, Regione Gonzole 10, 10043 Orbassano, Italy; nima.namarvari@unito.it (N.N.); francesco.franco@unito.it (F.F.)

**Keywords:** juvenile idiopathic arthritis, quality of life, patient-reported outcomes, pediatric rheumatology

## Abstract

*Background and Objectives*: Despite recent advancements in treatment, children with juvenile idiopathic arthritis (JIA) continue to experience poor health-related quality of life, and data on patient and parent satisfaction with disease management remain limited. Thus, this cross-sectional study aimed to explore the factors influencing parental satisfaction with their child’s JIA care, using the juvenile arthritis parent acceptable symptom state (JA-PASS). *Materials and Methods*: Parents of 63 children (43 females and 20 males; mean age 12.2 ± 3.7 years) diagnosed with JIA completed the Juvenile Arthritis Multidimensional Assessment Report (JAMAR). The study analyzed JAMAR responses, along with demographic data, disease duration and activity, and current medication use, to identify clinical factors that influence JA-PASS. *Results*: According to the JAMAR, 55.6% of parents expressed satisfaction with their child’s current condition. In a multiple regression analysis, significant factors influencing JA-PASS included medication side effects (*p* = 0.033), current disease activity (*p* = 0.009), and the psychosocial well-being rating in the JAMAR questionnaire (*p* = 0.048). *Conclusions*: JA-PASS should be integrated into patient assessment protocols, as it provides valuable insight into parents’ perceptions of disease progression and effectiveness of therapeutic interventions.

## 1. Introduction

Juvenile idiopathic arthritis (JIA) is a clinically heterogeneous group of chronic diseases characterized by joint inflammation of unknown etiology, with symptoms persisting for at least six weeks and an onset before the age of 16 years [1]. It is the most common rheumatic disorder in children, affecting approximately 16 to 50 children per 100,000 in high-income countries [2]. In Italy, around 10,000 children are affected by the disease [3].

The International League of Associations for Rheumatology (ILAR) classifies JIA into seven subtypes: systemic, polyarticular (rheumatoid factor [RF] negative or positive), oligoarticular, enthesitis-related (ERA), psoriatic, or undifferentiated [4]. Despite variations in disease course, all JIA subtypes share the hallmark of persistent synovial inflammation, leading to joint stiffness, swelling, and pain. These symptoms significantly impair daily activities and productivity compared to healthy peers [5]. In severe cases, this inflammatory process may result in permanent damage to articular cartilage and bone, leading to substantial physical disability [5].

The primary goal in managing JIA is to achieve and sustain disease remission, or at least minimal disease activity [6]. However, this remains challenging despite significant advances in drug therapy, especially with the introduction of advanced disease-modifying antirheumatic drugs (DMARDs) that target specific immune system pathways [7,8]. Consequently, the disease’s symptoms and activity can greatly impact the health-related quality of life (HRQoL) of affected children [9,10]. Furthermore, medication side effects can contribute to additional pain and stress, further deteriorating the patient’s overall well-being [11].

In this context, parent-/child-reported outcomes (PCROs) provide valuable insights into the disease’s course and the perceived benefits of therapeutic interventions, aiding medical decision-making and improving patient care [12]. Among the available tools, the Juvenile Arthritis Multidimensional Assessment Report (JAMAR) questionnaire captures key PCROs in JIA, including overall well-being, functional status, HRQoL, and pain intensity [13,14]. It also assesses satisfaction with the current state of the illness through a binary final question [15,16]. The concept of patient acceptable symptom state (PASS) is particularly relevant, as it reflects the symptom threshold beyond which health status is considered satisfactory by children with JIA and their parents (JA-CASS and JA-PASS, respectively) [17].

PASS is a relatively recent concept that captures the patient’s perception of being well [18], and it has been increasingly adopted across various medical disciplines, including orthopedics and rheumatology [19,20]. Its ease of use and strong association with healthcare outcomes make it a a valuable tool for evaluating treatment response on an individual level. As such, PASS should be considered a meaningful treatment target when balancing the benefits and risks of different therapeutic interventions [17]. However, PASS estimates may vary across different populations, even among individuals with the same condition [20].

While the impact of JIA on HRQoL and physical function has been extensively studied [6,21,22], there is limited research in the literature on patient and parent satisfaction [23]. Therefore, the objective of this cross-sectional study was to identify the clinical variables associated with JA-PASS using the JAMAR questionnaire in a specialized center in northern Italy.

## 2. Materials and Methods

### 2.1. Study Design and Patient Selection

Consecutive children diagnosed with JIA were recruited from those under follow-up at the Rheumatology Unit of Rivoli Hospital (Italy) between January 2020 and July 2022. Eligible patients were under 16 years of age, of both sexes, and diagnosed with any JIA subtype according to the ILAR criteria [4]. Patients with cognitive impairment and chronic conditions that could affect HRQoL or pain perception were excluded. They were also excluded if their parents lacked sufficient fluency in Italian to understand and complete the JAMAR questionnaire. Written informed consent was provided by parents/guardians prior to enrollment, and assent was obtained from participants as appropriate for their age. The study was conducted in accordance to the principles of the Helsinki Declaration, as revised in 2013, and was approved by the Institutional Ethics Committee. Reporting complied with the STROBE guidelines for cross-sectional studies.

### 2.2. Instrument

A parent (mother or father) or legal guardian of each enrolled patient was asked to complete the cross-culturally adapted and validated Italian parent version of the JAMAR questionnaire prior to a clinical visit [24]. The JAMAR is a multidimensional assessment tool consisting of 15 sections. For the purposes of this study, in addition to the question on the degree of satisfaction with the present child’s disease status (with yes or no response), corresponding to the JA-PASS, data from 7 of the 15 original domains were utilized. They included the following: (1) physical function measured through the 15-item Juvenile Arthritis Functionality Scale (JAFS) [25], which rates the child’s ability to perform daily tasks on a Likert scale (from 0 = without difficulty to 3 = unable to do), with total score ranging from 0 to 45; (2) HRQoL assessment using the 10-item Pediatric Rheumatology Quality of Life Scale (PRQL) questionnaire [26], organized into two subdimensions [physical health (PhH) and psychosocial health (PsH)]. Each consists of 5 items, with a scoring total from 0 to 15, with higher scores indicating worse HRQoL; (3) pain intensity graded using a visual analog scale (VAS), with scores ranging from 0 (no pain) to 10 (worst scenario) [27]; (4) degree and duration of morning stiffness; (5) perception of disease activity graduated from 0 (no activity) to 10 (maximum activity) on a VAS scale; (6) assessment of well-being on the VAS (from 0 = very well to 10 = very bad); and (7) degree of school difficulties caused by the disease, including difficulty attending school, inability to sit for extended periods, disagreement with teachers, or problems performing well.

### 2.3. Additional Information

The following information was collected from the patient’s medical records: demographic characteristics, disease-related variables such as JIA category, positivity for antinuclear antibody (ANA), RF, presence of human leukocyte antigen (HLA) B27 (HLA-B27) and B51 (HLA-B51), age at disease onset, age at visit, disease duration, and disease-activity parameters including the number of active and limited joints as assessed by the physician. Disease status (remission, continued activity, or flare) and current use of medication (type of medication, treatment adhesion, side effects) were also recorded. The involvement of the temporomandibular joint was assessed by an experienced dentist.

### 2.4. Data Analysis

The primary outcome of the study was satisfaction with child’s disease status. Thus, parents/caregivers were divided in two groups based on positive/negative JA-PASS according to the JAMAR questionnaire.

Data were all anonymously collected, analyzed, and published as aggregates. Quantitative data were reported using the mean value and standard deviation or the median and interquartile range (IQR). Categorical data were summarized using absolute and relative frequencies. The normality of continuous variables was verified with the Shapiro–Wilk test. For the comparison of quantitative variables between the two groups, the Student’s *t*-test for independent samples or the non-parametric Mann–Whitney test were applied according to their normal/non-normal distribution. Categorical variables were analyzed using the chi-square test or Fisher’s exact test, as appropriate.

A multiple logistic regression model was built to identify predictors of positive JA-PASS using a stepwise backward selection procedure. All potential explanatory variables that were significantly associated with the outcome in univariate models were entered in the multivariate logistic regression analysis. The results were presented as odds ratio (OR) with 95% confidence interval (CI). Finally, the area under the receiver operating characteristic curve (AUC) of the best-fitting model was used as an indicator of its predictive ability.

A *p*-value of 5% was set for statistical significance. All statistical tests were 2-sided and were performed using SPSS statistical package version 28 (IBM, Chicago, IL, USA).

## 3. Results

### 3.1. Patient Characteristics

**Figure 1:**
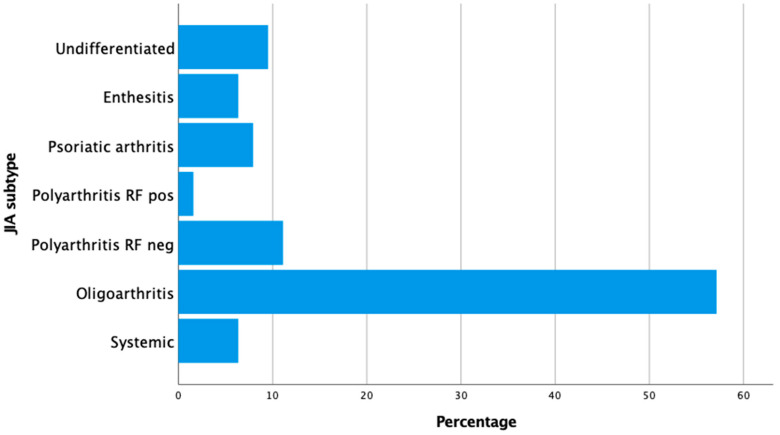
Frequency distribution by juvenile idiopathic arthritis subtype.

Sixty-three consecutive children with JIA, with a mean age of 12.2 ± 3.7 years, were enrolled in the study. Of these, 43 (68.3%) were girls. A parent of each patient, consisting of 44 mothers and 19 fathers, completed the JAMAR questionnaire. As reported in Figure 1, the majority of children (57.1%) had oligoarticular JIA, followed by polyarticular (12.7%) and psoriatic disease subtypes (7.9%).

The child’s demographic and clinical characteristics, along with disease-related measures and JAMAR domain scores, are presented in Table 1. The mean disease duration was 6.0 ± 3.8 years. The presence of ANA was found in 46% (n = 29) of individuals, while 6.3% (n = 4) tested positive for HLA-B27. The median active joint count was 0 (IQR 0–2). Fever and skin rashes were observed in only two children.

Methotrexate and tumor necrosis factor inhibitor agents were the most commonly used drugs, either as monotherapy or in combination. Nearly half of the patients experienced side effects, with nausea, gastritis, and vomiting being the most frequently reported. However, all parents declared children adhering to their prescribed medication regimen.

### 3.2. Assessment of JA-PASS Status

The majority of the parents (55.6%) were satisfied with their child’s current disease condition according to the JA-PASS. Demographic and disease-related characteristics of positive/negative JA-PASS are separately shown in Table 2.

A negative JA-PASS was significantly associated with specific JIA types, with a higher prevalence of ERA, systemic arthritis, and polyarticular arthritis (*p* = 0.004). However, no associations were found between the JA-PASS and patients’ demographic characteristics, although a higher proportion of fathers reported satisfaction than mothers (*p* = 0.026). The duration of the disease or age at diagnosis appeared to have no impact.

**Figure 2:**
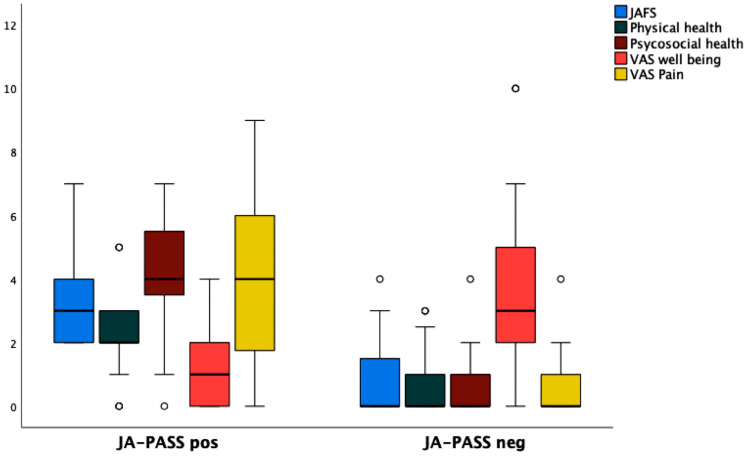
Parents’ score in the JAMAR domains grouped by positive or negative JA-PASS (box and whisker plots).

Conversely, active disease state, current medication intake, and drug side effects were reported more frequently among participants whose parents expressed dissatisfaction with the child’s JIA condition. Regarding the JAMAR domains, as displayed in Figure 2, statistically significant differences were found between parents with positive and negative JA-PASS in terms of functional ability, physical and psychosocial health, pain level, and child’s current well-being. Parents of children with a negative JA-PASS reported worse scores across all these items (all *p* < 0.001).

With the exception of four cases, all parents in the positive JA-PASS group rated the severity of disease activity as 0, whereas none of the parents in the negative JA-PASS group reported a severity score of 0 (*p* < 0.001).

### 3.3. Variables Associated with JA-PASS

In the stepwise logistic regression model (Table 3), the child’s current illness status (OR = 0.024, *p* = 0.009), the presence of at least one medication side effect (OR = 0.054, *p* = 0.033), and a higher psychosocial well-being rating on the JAMAR questionnaire (OR = 0.443, *p* = 0.048) were identified as the most significant independent variables associated with a negative JA-PASS. Other predictors that were statistically significant in the univariate analysis were no longer significant contributors to the model. The AUC value for the final regression model was 0.97 (95% CI 0.95–0.99).

## 4. Discussion

Juvenile idiopathic arthritis is a clinically heterogeneous disease, characterized by wide variability in presentation and disease course, often leading to significant impairment in quality of life [1]. Its impact is influenced not only by physical symptoms but also by parents’ perceptions of disease progression and treatment effectiveness [5]. This cross-sectional study aimed to identify factors influencing parental satisfaction with the treatment of JIA in a cohort of pre-adolescent children. Key contributors to parental satisfaction included the child’s current disease activity, medication side effects, and psychosocial well-being, as measured by the JAMAR questionnaire. A stepwise logistic regression model identified these variables as significant predictors, yielding an AUC of 0.97.

Assessing overall quality of life in children with JIA requires consideration of multiple complex and interrelated factors. The JAMAR is regarded as a gold-standard tool for evaluating these complexities, as it allows for a comprehensive assessment of the child’s symptoms, addressing both physical and psychosocial domains [15]. Additionally, the JA-PASS score, which is a component of the broader JAMAR tool, assesses parental perspectives on treatment effectiveness and whether they perceive their child’s current health status as acceptable [17]. Although it consists of a global dichotomized simple question, it has a good construct/discriminative validity, yielding a positive association with disease activity and severity [17,23,28]. Nevertheless, aspects such as emotional burden, coping mechanisms, or dissatisfaction with specific elements of care may not be fully addressed by the current structure of the questionnaire. These limitations should be taken into account in future research efforts aimed at improving the JAMAR questionnaire.

This study, conducted on 63 children in Italy, predominantly female, found oligoarticular JIA to be the most common disease subtype. A median active joint count of zero, along with a high proportion of children in remission at the time of evaluation, indicates a relatively low overall disease burden. These findings suggest that the study population is well-positioned to achieve and sustain an optimal HRQoL.

In line with previous studies [17,23], more than half of the parents (55.6%) expressed satisfaction with their child’s disease condition, despite significant side effects associated with standard medications such as methotrexate and tumor necrosis factor inhibitors [1,9]. Nevertheless, adherence to prescribed treatments remained high, even in the presence of common adverse effects such as nausea, gastritis, and vomiting. This highlights the substantial burden of managing treatment-related side effects, which adds to the challenges faced by families coping with JIA. While parents acknowledge that no treatment is without risk and appreciate the importance of therapy, their dissatisfaction often arises from adverse reactions that are perceived as intolerable. Addressing these concerns through a more individualized approach to JIA management, which carefully balances the benefits of treatment with its potential adverse effects, may enable healthcare providers to improve the overall treatment framework.

A key finding in this study is the strong influence of disease activity on parental satisfaction. Dissatisfaction was significantly associated with more severe disease presentations, particularly among patients with ERA, systemic arthritis, and polyarticular JIA. These subtypes are often linked to higher disease activity and greater functional impairment, which likely contribute to increased parental concern [1]. Consequently, a reduction in disease activity is strongly correlated with improved parental perceptions of treatment effectiveness and their child’s overall well-being. Notably, when children with JIA achieve disease remission, parents report higher satisfaction with care and enhanced quality of life for their children [20]. These findings underscore the importance of achieving and sustaining disease remission as the central goal in JIA treatment [21].

Furthermore, the present study further highlights the significant role of psychosocial well-being in shaping parental satisfaction. Parents of children who reported better psychosocial scores on the JAMAR questionnaire were more likely to express satisfaction with treatments. Although often underrecognized, the psychosocial impact of JIA emerges as a critical factor influencing the perceptions of both children and their families. Importantly, psychosocial stress is not only a consequence, but also a potential contributor to worse disease outcomes through mechanisms involving poor treatment adherence or amplifying symptoms’ perception. Thus, effective management strategies should address not only physical symptoms, but also promote psychosocial well-being, as this is essential for the child’s overall health and also for improving parental satisfaction with care.

The reliability of the assessment tools is supported by the finding that patients’ demographic characteristics did not influence JA-PASS outcomes, suggesting that the tool is not biased by such factors. Interestingly, a higher proportion of fathers reported satisfaction compared to mothers (*p* = 0.026), highlighting the potential need for a parent-specific approach in future research. Previous studies have identified gender differences with respect to caregiving roles and illness perception. Mothers usually assume the role of primary caregivers and report significantly higher levels of parenting stress and depressive symptoms compared with fathers when caring children with chronic or rare diseases [29,30,31]. Moreover, mothers tend to focus more on the negative aspects of their children’s chronic illnesses, whereas fathers are generally more accepting of their child’s disease conditions [29,32]. Thus, developing a tailored questionnaire that independently captures the perspectives of both mothers and fathers could provide a more nuanced and comprehensive understanding of parental experience with JIA care.

While this study provides valuable insights into the factors influencing parental satisfaction with JIA care, some limitations should be acknowledged. First, the study was conducted on a relatively small, single-center, cohort of Italian children, all receiving care at the same hospital. Larger, multicenter studies are needed to validate these findings and to explore potential country variations. Additionally, longitudinal studies that follow patients over time would provide a deeper understanding of how parental satisfaction evolves in relation to changes in disease activity and treatment plans.

Furthermore, this analysis was limited to the items included in the JAMAR, excluding factors such as fatigue, which is a common and burdensome issue not captured by the tool. Future research should also explore the influence of additional factors, such as socio-economic status and healthcare access, on parental satisfaction. These variables may affect families’ ability to manage the disease, adhere to treatment protocols, and impact the quality of life for both children and parents [33]. Finally, exploring interventions aimed at improving psychosocial well-being and mitigating treatment-related side effects could help identify strategies that enhance overall treatment outcomes and satisfaction.

## 5. Conclusions

The present study shows that the JA-PASS provides valuable insight into parents’ perceptions of disease progression and effectiveness of therapeutic interventions. Thus, it should be incorporated into patient assessment protocols in both routine clinical practice and clinical trials.

Despite the improvement in functional outcomes achieved in the last few years, about 44% of parents are still unsatisfied with the current state of their child’s disease. The factors most strongly associated with parental dissatisfaction are child’s current disease activity, psychosocial well-being, and medication side effects. These areas highlight key opportunities for improving medical management. If left unaddressed, parental dissatisfaction may contribute to therapy nonadherence and increased distress in patients. Thus, incorporating the questionnaire into medical decision-making could play a significant role in enhancing the quality of care for children with JIA. Although pain was not significantly associated with parents’ dissatisfaction in the regression logistic model, the high VAS scores for pain perception suggest that there is also a need for improvement in children’s pain control.

## Figures and Tables

**Table 1:** Demographic and disease severity characteristics of children with JIA at enrolment.

Variables	N = 63
Patient’s characteristics	
Sex female, n (%)	43 (68.3)
JIA subtypes, n (%)	
Oligoarthritis	36 (57.1)
Polyarthritis, RF positive	1 (1.6)
Polyarthritis, RF negative	7 (11.1)
Psoriatic arthritis	5 (7.9)
Enthesitis-related arthritis	4 (6.3)
Systemic arthritis	4 (6.3)
Undifferentiated arthritis	6 (9.6)
ANA positive, n (%)	29 (46.0)
HLA-B27 positive, n (%)	4 (6.3)
HLA-B51 positive, n (%)	1 (1.6)
Age at disease onset, mean (SD), years	6.0 (3.8)
Disease duration, mean (SD), years	6.1 (3.3)
Drug therapy	
Medication intake, n (%)	40 (63.5)
Type of medication, n (%)	
NSAIDS	6 (9.5)
Glucocorticoids	4 (6.3)
bDMARDS (TNFi)	10 (15.9)
MTX	21 (33.3)
MTX + TNFi	6 (9.5)
bDMARDS (non-TNFi)	2 (3.2)
None	23 (36.5)
Side effects, n (%)	27 (47.9)
Active joints, median (IQR)	0 (0–2)
TMJ pain, n (%)	25 (39.7)
TMJ stiffness, n (%)	24 (38.1)
Morning stiffness over the past week by JAMAR report, n (%)	19 (30.2)
JAFS, median (IQR)	2.0 (0.0–3.0)
PhH, median (IQR)	1.0 (0.0–2.0)
PsH, median (IQR)	2.0 (0.0–4.0)
VAS pain, median (IQR)	1.5 (0.0–4.3)
VAS disease activity, median (IQR)	1.0 (0.0–5.0)
VAS well-being, median (IQR)	2.0 (1.0–3.6)
School difficulties, n (%)	17 (27.0)

JIA: juvenile idiopathic arthritis; ANA: antinuclear antibody; RF: rheumatoid factor; NSAIDS: non-steroidal inflammatory drugs; MTX: methotrexate; bDMARDS: biological disease-modifying antirheumatic drugs; TNFi: tumor necrosis factor inhibitor; SD: standard deviation; IQR: interquartile range; TMJ: temporomandibular joint; JAFS: Juvenile Arthritis Functionality Scale; PhH: physical health; PsH: psychological health; VAS: visual analog scale.

**Table 2:** Demographic and disease severity characteristics stratified by JA-PASS.

Variables	JA-PASS Positive (n = 35)	JA-PASS Negative (n = 28)	*p* Value
Father, n (%)	15 (42.9)	4 (14.3)	0.026
Patient’s characteristics			
Sex female, n (%)	21 (60.0)	22 (78.6)	0.173
JIA subtypes, n (%)			0.004
Oligoarthritis	27 (77.1)	9 (32.2)	
Poliarthritis	2 (5.7)	6 (21.4)	
Systemic arthritis—ERA	2 (5.7)	6 (21.4)	
Others	4 (11.5)	7 (25.0)	
Age at visit, mean (SD), years	11.7 (3.5)	12.6 (4.0)	0.321
Age at disease onset, mean (SD), years	5.3 (3.4)	6.9 (4.1)	0.090
Disease duration, mean (SD), years	5.7 (3.7)	6.3 (3.0)	0.436
Drug therapy			
Medication intake, n (%)	17 (48.6)	23 (82.1)	0.015
Side effects, n (%)	3 (8.6)	24 (85.7)	<0.001
Disease severity			
Current disease state, n (%)			<0.001
Disease activity	2 (5.7)	19 (67.9)	
Relapse	0 (0.0)	4 (14.3)	
Remission	33 (94.3)	5 (17.9)	
Active joints, median (IQR)	0.0 (0.0–0.0)	1.0 (0.0–2.0)	<0.001
TMJ pain, n (%)	9 (25.7)	16 (57.1)	0.011
TMJ stiffness, n (%)	5 (14.3)	19 (67.9)	<0.001
Morning stiffness by JAMAR report, n (%)	10 (28.6)	9 (32.1)	0.756
JAFS, median (IQR)	0.0 (0.0–1.5)	3.0 (2.0–4.0)	<0.001
PhH, median (IQR)	0.0 (0.0–1.0)	2.0 (2.0–3.0)	<0.001
PsH, median (IQR)	0.0 (0.0–1.3)	4.0 (3.3–5.8)	<0.001
VAS pain, median (IQR)	0.0 (0.0–1.0)	4.0 (1.6–6.0)	<0.001
VAS disease activity, median (IQR)	0.0 (0.0–0.0)	5.0 (2.1–7.0)	<0.001
VAS well-being, median (IQR)	3.0 (2.0–5.0)	1.0 (0.0–2.0)	<0.001
School difficulties, n (%)	3 (8.6)	14 (50.0)	0.001

JIA: juvenile idiopathic arthritis; SD: standard deviation; IQR: interquartile range; TMJ: temporomandibular joint; JAFS: Juvenile Arthritis Functionality Scale; PhH: physical health; PsH: psychological health; VAS: visual analog scale.

**Table 3:** Multiple logistic regression model with a positive JA-PASS status as dependent variable (R^2^ = 0.897).

Variables	Odds Ratio (95% CI)	*p* Value
Intercept	124.399	
Current disease state (activity or relapse vs. remission)	0.024 (0.001–0.389)	0.009
Psychosocial well-being, points	0.443 (0.199–0.984)	0.048
Presence of drug side effects (yes vs. no)	0.054 (0.004–0.785)	0.033

## Data Availability

The data presented in this study are available on request from the corresponding author due to ethical restriction.

## Abbreviations

The following abbreviations are used in this manuscript:

JIA	Juvenile idiopathic arthritis
ANA	Antinuclear antibody
RF	Rheumatoid factor
ERA	Enthesitis-related arthritis
NSAIDS	Non-steroidal inflammatory drugs
MTX	Methotrexate
bDMARDS	Biological disease-modifying antirheumatic drugs
TNFi	Tumoral necrosis factor inhibitor
SD	Standard deviation
IQR	Interquartile range
OR	Odds ratio
CI	Confidence interval
TMJ	Temporomandibular joint
JAFS	Juvenile Arthritis Functionality Scale
PhH	Physical health
PsH	Psychological health
VAS	Visual analog scale

## References

[1] Zaripova L.N., Midgley A., Christmas S.E., Beresford M.W., Baildam E.M., Oldershaw R.A. (2021). Juvenile idiopathic arthritis: From etiopathogenesis to therapeutic approaches. Pediatr. Rheumatol..

[2] Thierry S., Fautrel B., Lemelle I., Guillemin F. (2014). Prevalence and incidence of juvenile idiopathic arthritis: A systematic review. Jt. Bone Spine.

[3] Marini F., Falcini F., Stagi S., Fabbri S., Ciuffi S., Rigante D., Matucci Cerinic M., Brandi M.L. (2020). Study of vitamin D status and vitamin D receptor polymorphisms in a cohort of Italian patients with juvenile idiopathic arthritis. Sci. Rep..

[4] Petty R., Southwood T.R., Manners P., Baum J., Glass D.N., Goldenberg J., He X., Maldonado-Cocco J., Orozco-Alcala J., Prieur A.-M. (2004). International League of Associations for Rheumatology classification of juvenile idiopathic arthritis: Second revision, Edmonton, 2001. J. Rheumatol..

[5] Onel K., Rumsey D.G., Shenoi S. (2021). Juvenile idiopathic arthritis treatment updates. Rheum. Dis. Clin. N. Am..

[6] Martini A. (2019). Are there new targets for juvenile idiopathic arthritis?. Semin. Arthritis Rheum..

[7] Welzel T., Winskill C., Zhang N., Woerner A., Pfister M. (2021). Biologic disease modifying antirheumatic drugs and Janus kinase inhibitors in pediatric rheumatology—What we know and what we do not know from randomized controlled trials. Pediatr. Rheumatol. Online J..

[8] Selvaag A.M., Aulie H.A., Lilleby V., Flato B. (2016). Disease progression into adulthood and predictors of long-term active disease in juvenile idiopathic arthritis. Ann. Rheum. Dis..

[9] Doeleman M.J.H., de Roock S., Buijsse N., Klein M., Bonsel G.J., Seyfert-Margolis V., Swart J.F., Wulffraat N.M. (2021). Monitoring patients with juvenile idiopathic arthritis using health-related quality of life. Pediatr. Rheumatol..

[10] Chédeville G., McGuire K., Cabral D.A., Shiff N.J., Rumsey D.G., Proulx-Gauthier J.P., Schmeling H., Berard R.A., Batthish M., Soon G. (2022). Parent-reported medication side effects and their impact on health-related quality of life in children with juvenile idiopathic arthritis. Arthritis Care Res..

[11] Hassan S., Wamithi S., Riang’a R.M., Migowa A. (2025). Experiences among parents caring for children with juvenile idiopathic arthritis at a tertiary referral hospital in Kenya. Front. Pediatr..

[12] Lavallee D.C., Chenok K.E., Love R.M., Petersen C., Holve E., Segal C.D., Franklin P.D. (2016). Incorporating patient-reported outcomes into health care to engage patients and enhance care. Health Aff..

[13] Taxter A.J., Wileyto E.P., Behrens E.M., Weiss P.F. (2015). Patient-reported outcomes across categories of juvenile idiopathic arthritis. J. Rheumatol..

[14] Esi M.M., Carle A.C. (2020). Measures of health status and quality of life in juvenile idiopathic arthritis. Arthritis Care Res..

[15] Filocamo G., Consolaro A., Schiappapietra B., Dalprà S., Lattanzi B., Magni-Manzoni S., Ruperto N., Pistorio A., Pederzoli S., Civino A. (2011). A new approach to clinical care of juvenile idiopathic arthritis: The Juvenile Arthritis Multidimensional Assessment Report. J. Rheumatol..

[16] Bovis F., Consolaro A., Pistorio A., Garrone M., Scala S., Patrone E., Rinaldi M., Villa L., Martini A., Ravelli A. (2018). Cross-cultural adaptation and psychometric evaluation of the Juvenile Arthritis Multi-Dimensional Assessment Report (JAMAR) in 54 languages across 52 countries: Review of the general methodology. Rheumatol. Int..

[17] Filocamo G., Consolaro A., Schiappapietra B., Ruperto N., Pistorio A., Solari N., Pederzoli S., Verazza S., Martini A., Ravelli A. (2012). Parent and child acceptable symptom state in juvenile idiopathic arthritis. J. Rheumatol..

[18] Tubach F., Dougados M., Falissard B., Baron G., Logeart I., Ravaud P. (2006). Feeling good rather than feeling better matters more to patients. Arthr. Rheum..

[19] De Wolff L., Vissink A., van Nimwegen J.F., van Zuiden G., Mossel E., Olie L., Stel A.J., Delli K., Verstappen G.M., Kroese F.G.M. (2022). Patient Acceptable Symptom State (PASS) in patients with primary Sjögren’s syndrome in daily clinical practice. Clin. Exp. Rheumatol..

[20] Daste C., Abdoul H., Foissac F., Lefèvre-Colau M.M., Poiraudeau S., Rannou F., Nguyen C. (2022). Patient acceptable symptom state for patient-reported outcomes in people with non-specific chronic low back pain. Ann. Phys. Rehabil. Med..

[21] Vanoni F., Suris J.C., Scheven-gete A.V., Fonjallaz B., Hofer M. (2016). The difference of disease perception by juvenile idiopathic arthritis patients and their parents: Analysis of the JAMAR questionnaire. Pediatr. Rheumatol..

[22] Consolaro A., Ruperto N., Filocamo G., Lanni S., Bracciolini G., Garrone M., Scala S., Villa L., Silvestri G., Tani D. (2012). Pediatric Rheumatology International Trials Organization (PRINTO). Seeking insights into the EPidemiology, treatment and Outcome of Childhood Arthritis through a multinational collaborative effort: Introduction of the EPOCA study. Pediatr. Rheumatol..

[23] Del Giudice E., de Roock S., Vastert S.J., Wulffraat N.M., Swart J.F., van Dijkhuizen E.H.P. (2023). Patients’ and parents’ satisfaction to improve patient care in JIA: Factors determining acceptable symptom state measured with JAMAR. Rheumatology.

[24] Consolaro A., Bovis F., Pistorio A., Cimaz R., De Benedetti F., Miniaci A., Corona F., Gerloni V., Martino S., Pastore S. (2018). The Italian version of the Juvenile Arthritis Multidimensional Assessment Report (JAMAR). Rheumatol. Int..

[25] Balay-Dustrude E., Shenoi S. (2023). Current validated clinical and patient reported disease outcome measures in juvenile idiopathic arthritis. Open Access Rheumatol..

[26] Ford J.P., Soriano E.R., Andreu M. (2025). Evaluation of the psychometric properties of the Functional Ability Scale in children with juvenile idiopathic arthritis. Reumatol. Clin. (Engl. Ed.).

[27] Filocamo G., Davi S., Pistorio A., Bertamino M., Ruperto N., Lattanzi B., Consolaro A., Magni-Manzoni S., Galasso R., Varnier G.C. (2010). Evaluation of 21-numbered circle and 10-centimeter horizontal line visual analog scales for physician and parent subjective ratings in juvenile idiopathic arthritis. J. Rheumatol..

[28] Salaffi F., Carotti M., Gutierrez M., Di Carlo M., De Angelis R. (2015). Patient acceptable symptom state in self-report questionnaires and composite clinical disease index for assessing rheumatoid arthritis activity: Identification of cut-off points for routine care. BioMed Res. Int..

[29] Cardinali P., Migliorini L., Rania N. (2019). The caregiving experiences of fathers and mothers of children with rare diseases in Italy: Challenges and social support perceptions. Front. Psychol..

[30] Chu S.-Y., Wen C.-C., Weng C.-Y. (2022). Gender differences in caring for children with genetic or rare diseases: A mixed-methods study. Children.

[31] Matthews A., Lenz K.R., Peugh J., Copps E.C., Peterson C.M. (2018). Caregiver burden and illness perceptions in caregivers of medically hospitalized youth with anorexia nervosa. Eat. Behav..

[32] Knafl K., Zoeller L. (2000). Childhood chronic illness: A comparison of mothers’ and fathers’ experiences. J. Fam. Nurs..

[33] Kraft P., Kraft B. (2021). Explaining socioeconomic disparities in health behaviors: A review of biopsychological pathways involving stress and inflammation. Neurosci. Biobehav. Rev..

